# ConsensusPathDB 2022: molecular interactions update as a resource for network biology

**DOI:** 10.1093/nar/gkab1128

**Published:** 2021-11-25

**Authors:** Atanas Kamburov, Ralf Herwig

**Affiliations:** R&D Digital Technologies Department, Bayer AG, Berlin 13353, Germany; Department of Computational Molecular Biology, Max Planck Institute for Molecular Genetics, Berlin 14195, Germany

## Abstract

Molecular interactions are key drivers of biological function. Providing interaction resources to the research community is important since they allow functional interpretation and network-based analysis of molecular data. ConsensusPathDB (http://consensuspathdb.org) is a meta-database combining interactions of diverse types from 31 public resources for humans, 16 for mice and 14 for yeasts. Using ConsensusPathDB, researchers commonly evaluate lists of genes, proteins and metabolites against sets of molecular interactions defined by pathways, Gene Ontology and network neighborhoods and retrieve complex molecular neighborhoods formed by heterogeneous interaction types. Furthermore, the integrated protein–protein interaction network is used as a basis for propagation methods. Here, we present the 2022 update of ConsensusPathDB, highlighting content growth, additional functionality and improved database stability. For example, the number of human molecular interactions increased to 859 848 connecting 200 499 unique physical entities such as genes/proteins, metabolites and drugs. Furthermore, we integrated regulatory datasets in the form of transcription factor–, microRNA– and enhancer–gene target interactions, thus providing novel functionality in the context of overrepresentation and enrichment analyses. We specifically emphasize the use of the integrated protein–protein interaction network as a scaffold for network inferences, present topological characteristics of the network and discuss strengths and shortcomings of such approaches.

## INTRODUCTION

Modern biomedical experiments, for example the generation of cell atlases (1) or patient-derived disease-associated data (2), rely on high-throughput experiments such as sequencing, proteomics or genome-wide methylation experiments and agglomerate heterogeneous information from these diverse experiments. An important step in these workflows is the integration and interpretation of the data in the context of biological pathways and networks.

Biological networks typically consist of molecular interactions that have been experimentally measured by proteomics or genetic technologies, reported in the literature and assembled in interaction databases (3). However, such databases are often complementary in terms of content and tend to focus on one or a few types of interactions, while in biological processes all the different interaction types coexist in the cell. In order to obtain a global interaction map that reflects cell biology as comprehensively as possible, subject to the currently available interaction knowledge, many available interaction resources have to be used in parallel. Furthermore, it has been shown that the choice of a pathway database for analyzing a given dataset impacts results of gene enrichment analyses (4), which necessitates integration across such resources.

To this end, we have developed and maintained (since 2009) the ConsensusPathDB database that integrates different types of interactions from numerous resources into a seamless global network (5,6). In this network, physical entities (genes, proteins, protein complexes, metabolites, drugs, etc.) from different interaction sources are matched based on their accession numbers and interactions are matched based on the physical entities involved to reduce data redundancy. In ConsensusPathDB, we have agglomerated the content of 31 major public repositories on human molecular interactions of heterogeneous types as well as biochemical pathways resulting in one of the largest interactome maps available. Furthermore, separate instances of the database integrate the content of 16 mouse and 14 yeast interaction repositories, respectively. The web interface enables the research community to search and visualize complex subnetworks as well as to carry out overrepresentation/enrichment analysis and network analysis of lists of proteins, genes and metabolites (e.g. from large-scale experiments) in order to interpret experimental data. The integrated resources can be downloaded and used for network analysis, e.g. for network propagation-based methods. For example, the integrated protein–protein interaction (PPI) network of ConsensusPathDB has been recently benchmarked as one of the top-performing networks for disease gene identification among 21 comparable resources (7) and has been used for identifying network modules for monitoring drug actions across heterogeneous experiments (8).

In this 2022 update of ConsensusPathDB, we describe the novel content and functionality recently added to the database and the web interface, respectively. Notably, additional regulatory gene sets have been added to the overrepresentation and enrichment functionality comprising microRNA–, transcription factor– and enhancer–gene target sets, adding to the previously available options of using curated pathways, Gene Ontology (9) categories, network neighborhoods and protein complexes. Furthermore, we describe and characterize the new integrated PPI network that now comprises 522 618 human binary, physical interactions as a scaffold for network propagation analyses. ConsensusPathDB is freely accessible under http://consensuspathdb.org.

## ConsensusPathDB CONTENT UPDATE 2022

### Source databases and types of molecular interactions

Since our last report on ConsensusPathDB (5), the database has grown significantly in content (see Table 1 for human resources and Supplementary Table S1 for mouse and yeast resources). While the number of interaction source databases integrated in ConsensusPathDB stayed fairly constant (with the exception of DrugBank dropping out due to new access restrictions), its overall content increased significantly. Since the last report (5), the number of unique interactions stored in ConsensusPathDB has grown from 215 541 (version 25) to 859 848 human interactions in the current version 35 (+299%) mainly because the content of the included resources has grown. For human interactions, the integrated resources comprise 31 databases: BIND (10), BioCarta (11), Biogrid (12), CORUM (13), ChEMBL (14), DIP (15), EHMN (16), HPRD (17), HumanCyc (18), INOH (19), InnateDB (20), IntAct (21), KEGG (22), MINT (23), MIPS-MPPI (24), MatrixDB (25), NetPath (26), PDB (27), PDZBase (28), PID (29), PIGDB (30), PINdb (31), PharmGKB (32), PhosphoPOINT (33), PhosphoSitePlus (34), Reactome (35), SMPDB (36), SignaLink (37), SPIKE (38), TTD (39) and WikiPathways (40).

**Table 1.** Growth figures describing the increase in content of human interactions with respect to the last database publication in 2013 (5)

	Human		
Interaction type	2013: version 25 (# interactions)	2022: version 35 (# interactions)	Content growth (# interactions)
Protein–protein	155 855	616 304	460 449
Signaling or metabolic	20 682	25 046	4364
Gene regulatory	5658	18 912	13 254
Genetic	265	7936	7671
Drug–target	33 081	191 650	158 569
**Gene target sets**	**2013: version 25 (# sets)**	**2022: version 35 (# sets)**	**Content growth (# sets)**
Pathways	4601	5578	977
Protein complex-derived sets	39 685	244 987	205 302
miRNA–gene target	0	5474	5474
Transcription factor–gene target	0	800	800
Enhancer–gene target sets^a^	0	217 790	217 790

^a^ It should be noted that enhancer–gene target sets are highly redundant across different cell types.

**Figure 1.**
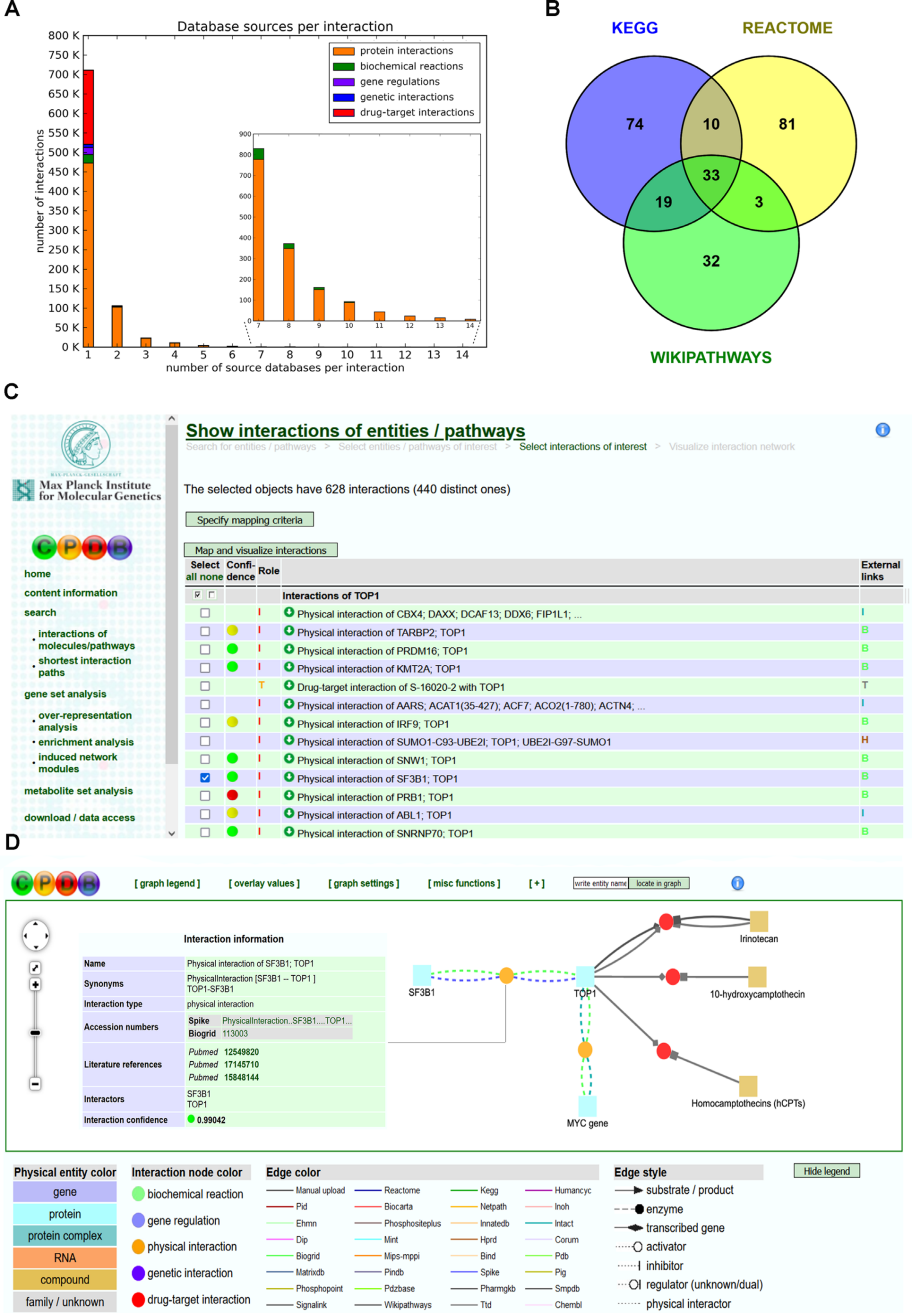
ConsensusPathDB content. (**A**) Number of interactions (*Y*-axis) shared by number of source databases (*X*-axis). The rightmost tail of the histogram is magnified. The colors within each bar represent the different interaction types. (**B**) Venn diagram of overlapping gene sets for the apoptosis pathway annotated by three prominent pathway databases: KEGG (pathway identifier: hsa04210), Reactome (R-HSA-109581) and WikiPathways (WP254). (**C**) Interaction display for the TOP1 (*DNA topoisomerase I*) gene. Binary interactions are scored for confidence and the confidence value is displayed by a traffic light symbol. Each interaction assigns a specific role to the molecule under study (e.g. ‘*I*’ interactor, ‘*T*’ target) and has an external link to the annotating source database. Interactions can be selected for further visualization. (**D**) Visualization of selected interactions of TOP1. Interactions are displayed with colored nodes indicating the interaction type and interacting molecules are displayed with colored squares indicating their type. Each connection represents a source database that has annotated the interaction. Clicking on each interaction (or molecule) displays further information about the interaction, the confidence score and supporting publications.

A major motivation for providing a meta-resource for molecular interactions is the complementarity of the different source databases. Analysis of the total number of source databases per interaction in ConsensusPathDB shows that the respective distribution is right-skewed, with most of the interactions (83%) originating from a single source database (Figure 1A). These results show that currently available databases are highly complementary and, importantly, that the integrated interaction map present in ConsensusPathDB has not saturated yet. The proportion of ‘unique’ interactions has even slightly increased compared to the 2013 version 25 of ConsensusPathDB where we observed 75% single-source interactions. This underlines the continued need for integration of interaction data in order to generate more complete interactomes. Most of the integrated interactions are protein interactions (72%) in the form of protein–protein binary interactions and protein complexes followed by drug–target interactions (22%) and biochemical reactions (3%) covering metabolic and signaling processes.

The individual databases have variable contribution to the overall content of ConsensusPathDB. The five largest resources are Biogrid (495 966 interactions), IntAct (162 374), ChEMBL (143 258), TTD (43 878) and HPRD (40 484). Also, the contribution to the novel interactions is largely driven by a few databases (Supplementary Figure S1), namely Biogrid (418 831 novel interactions; 53% of all novel interactions), IntAct (146 989; 19%), ChEMBL (143 258; 18%) and TTD (40 621; 5%), which account for 95% of all novel interactions.

In addition to molecular interactions, ConsensusPathDB contains 5578 pre-annotated pathway gene sets from 12 resources (KEGG, Reactome, WikiPathways, BioCarta, EHMN, HumanCyc, INOH, NetPath, PID, PharmGKB, SMPDB and SignaLink) available for overrepresentation and enrichment analyses. Complementarity of the annotation is also observable in this pathway content. For example, the gene sets for apoptosis signaling—one of the best studied pathways that has high relevance for cancer—provided by three prominent pathway databases [KEGG (hsa04210), Reactome (R-HSA-109581) and WikiPathways (WP254)] differ significantly, with 74% of the genes being unique to one of the databases, whereas only 13% of the genes are common for all three gene sets (Figure 1B). This ‘annotation bias’ can interfere with gene set enrichment analyses (4) and justifies using a variety of resources in such analysis workflows rather than only a single one.

### Integrative view on heterogeneous molecular interactions

The user can explore all integrated interactions of a molecule of interest in ConsensusPathDB through the web interface in several steps. In the first step, the molecule of interest can be retrieved by typing an identifier or molecule name. It is recommended to use official symbols, or UniProt or Ensembl identifiers in case of genes or proteins and KEGG, ChEBI or PubChem identifiers in the case of metabolites or drugs, since these are the basic annotation types of ConsensusPathDB. The database returns all entries that match with the search term. After selecting an entry, all interactions are listed with the gene/protein/metabolite of interest (Figure 1C). In case of binary PPIs, a confidence value is provided as a ‘traffic light’ icon in order to help structuring and reviewing the output. In the third step, interactions can be selected and visualized, enabling integrated views across heterogeneous interaction types and interaction resources (Figure 1D).

## ADDITION OF NOVEL REGULATORY GENE SETS

### Overrepresentation and enrichment analyses

Among the most widely used features of ConsensusPathDB are to perform enrichment analyses of user-defined lists of genes/proteins and metabolites with respect to pre-annotated pathways, GO categories, protein complexes and network neighborhoods as defined by the integrated PPI network. Overrepresentation analysis requires a simple list of gene/protein, metabolite, or identifiers and is computed with Fisher’s exact test. Enrichment analysis requires in addition numerical data from two different states for comparison (e.g. disease versus healthy state) and is computed with Wilcoxon’s rank sum test (41).

In this 2022 update, the basis for overrepresentation and enrichment analysis functionality has been extended to include regulatory gene sets in the form of microRNA–, transcription factor– and enhancer–gene target sets. As epigenetic studies and studies on post-transcriptional regulation have become frequent, we have thus addressed the need for analysis tools for such data based on gene sets defined by regulatory relationships in the current ConsensusPathDB version. Target gene sets were included from three different microRNA databases [TargetScan version 7.2 (42), miRTarBase version 8.0 (43) and miRDB version 6.0 (44)], one transcription factor–target interaction resource [TRRUST version 2 (45)] and one enhancer–target interaction resource [EnhancerAtlas version 2.0 (46)].

### Use case 1: exploring post-transcriptional regulation for cardiotoxicity

Recently, we have analyzed the effects of four anticancer therapies (doxorubicin, epirubicin, idarubicin and daunorubicin) in a human 3D cardiac microtissue model and identified a network of 142 proteins (Supplementary Data S1) that revealed common dynamic changes as measured with transcriptomic and proteomic time course experiments (8). It is well known that anthracyclines induce cardiotoxicity in patients, so we explored the ConsensusPathDB for information on disease pathways and metabolic processes that might be altered after drug treatment. Pathway overrepresentation with the set of 142 genes reveals multiple pathways related to cardiac diseases (Supplementary Data S1) such as ‘diabetic cardiomyopathy’ (KEGG hsa05415, *Q* = 8.02e−08) and ‘striated muscle contraction’ (Reactome R-HSA-390522, *Q* = 172e−07), among others, as well as metabolic pathways related to ‘electron transport’ (Reactome R-HSA-611105, *Q* = 721e−07; WikiPathways WP111, *Q* = 168e−05) and the ‘TCA cycle’ (Reactome R-HSA-1428517, *Q* = 887e−14; KEGG hsa00020, *Q* = 154e−10; EHMN TCA cycle, *Q* = 802e−08; HumanCyc PWY66-398, *Q* = 106e−07; WikiPathways WP78, *Q* = 146e−06) that account for mitochondrial dysfunction and relevant cardiotoxicity response pathways (47).

In addition to such pathway-based analyses, the ConsensusPathDB 2022 offers the analysis of microRNA–gene target sets. Overrepresentation analysis reveals 16 significantly enriched microRNA target sets (Supplementary Data S1; *Q* < 0.05). The top three candidates are those regulated by miR-615-3p, miR1-3p and miR92a-3p (*Q* = 0.00098) annotated from miRTarBase version 8.0. Literature evidence supports the role of these microRNAs for cardiac function and toxicity. For example, deletion of miR92a-3p has been reported to exert cardioprotective effects in mice (48). Most evidence has been reported for miR1-3p: in a recent study on human patients, it was found that different forms of cardiomyopathies had typical microRNA patterns and that miR1-3p was specific for hypertrophic cardiomyopathy where it was also correlating with clinical parameters such as left ventricular ejection function (49). More specifically, miR1-3p has been proposed as a biomarker for doxorubicin-induced cardiotoxicity after treatment of breast cancer patients, which accounts for the origin of the selected gene targets in the *in vitro* microtissue model (50). This use case exemplifies that the newly integrated gene sets can expand the knowledge from transcriptomics/proteomics-derived gene lists to post-transcriptional regulatory information.

### Use case 2: tissue-specific genes and transcription factors

Tissue-specific processes are often regulated by specific transcription factors and through the specific connections between transcription factors and their gene targets (51). Thus, enrichment analysis of transcription factor–target sets evaluated against user gene lists can provide valuable insights into regulatory mechanisms. We exemplified this by downloading the 100 most highly expressed genes across 226 liver samples as provided by the GTEx Consortium (52). Overrepresentation analysis with ConsensusPathDB reveals nine transcription factors significantly enriched by the top liver-expressed genes (Supplementary Data S2; *Q* < 0.05): *NR2F1*, *HNF4A*, *CEBPB*, *NR2F6*, *STAT3*, *HNF1A*, *CEBPA*, *PPARGC1A* and *TFCP2*. All detected factors play key roles in liver development and metabolism, for example hepatic nuclear factors, HNF1A and HNF4A (53), in liver disease pathology such as STAT3 (54) or in liver regeneration such as CEBPA and CEBPB (55). We conclude that, combining expression-based gene lists with transcription factor–target sets, can generate valuable hypotheses on transcriptional regulation of the system under study.

### Use case 3: putative enhancer regulation of the human cancer signaling network

ConsensusPathDB offers the possibility to interrogate lists of genes and proteins against enhancer–target gene sets derived from the EnhancerAtlas 2.0 database (46) that provides such information for 110 human cell lines. Enhancers are known to impact signaling pathways; for example, it has been shown that in cancer cells superenhancers promote oncogene expression and thus mediate dysregulation of several signaling pathways (56). In order to explore putative enhancer regulation of the cancer signaling network, we used 531 genes contained in the KEGG ‘Pathways in cancer’ network (ID 05200 N). Overrepresentation analysis yields 959 enriched enhancer–target sets (Supplementary Data S3; *Q* < 0.05). The corresponding enhancers are highly redundant across the different cell lines. Combining enhancers with the same gene targets yields 17 enhancer clusters that regulate 61 cancer genes. For example, *IL4* (interleukin 4), *IL5* and *IL13* genes are part of enhancer–target sets on chromosome 5 in many different cell lines. The genes are cytokines that are expressed in T helper type 2 cells and they mediate the escape of tumor cells in chronic infection. It has been shown that the expression of these genes is regulated by a genomic enhancer region that is located on chromosome 5 in the 3′ region of the RAD50 gene, which corresponds to the predicted enhancer regions in the different cell lines (57).

In order to cross-validate these 17 enhancer clusters and their role in cancer, we compared them against a recent pan-cancer analysis of enhancer expression from The Cancer Genome Atlas (TCGA) Consortium (58). In this study, enhancer patient gene expression was identified that has prognostic value for survival and 4 out of 17 enhancer clusters identified with the overrepresentation analysis indeed contained prognostic enhancers from the TCGA study. Thus, enhancer–target set enrichment can explore regulatory information inherent in user gene lists.

## INTEGRATING PPIs FOR NETWORK-BASED INFERENCES

### PPI confidence assessment and network characterization

**Figure 2.**
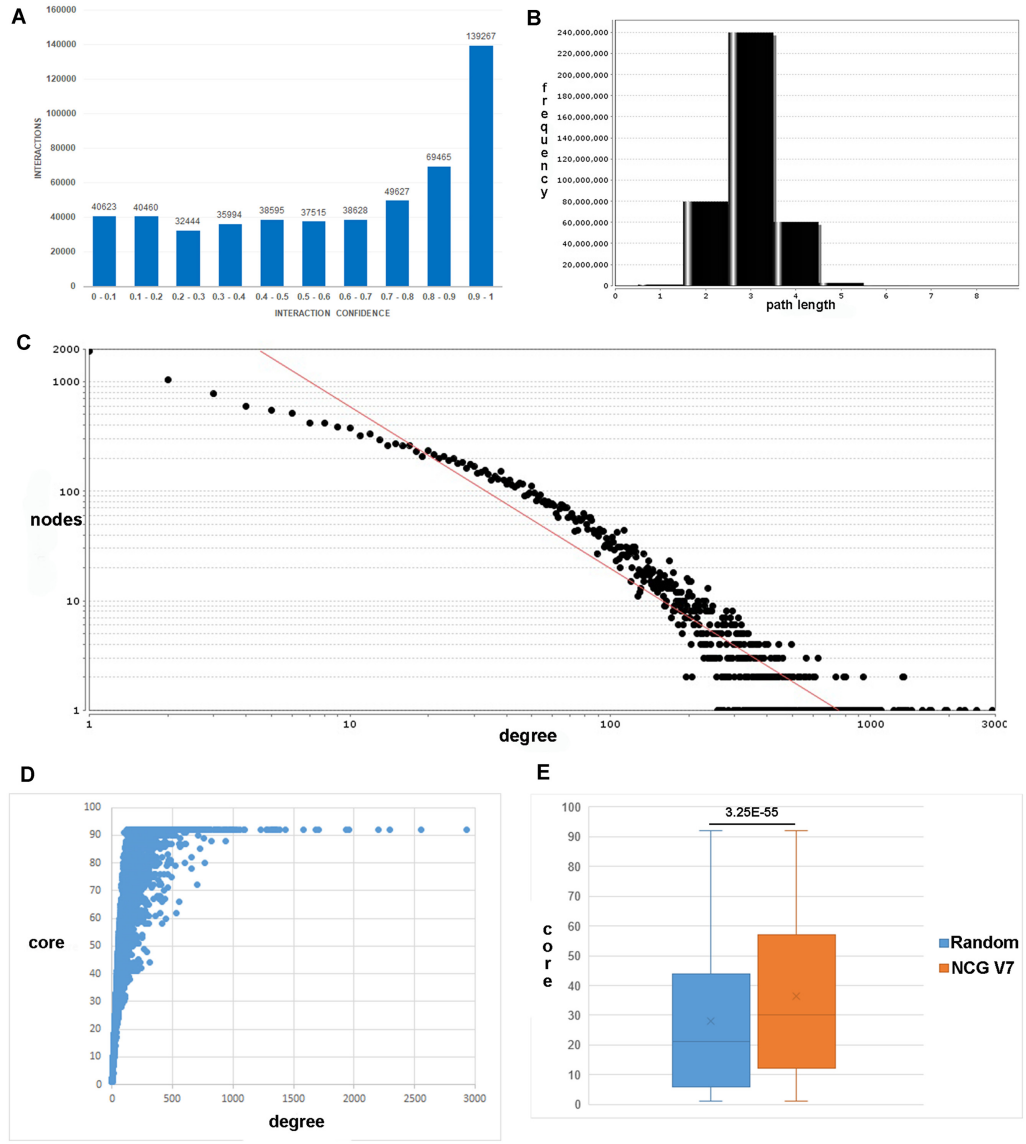
PPI network characteristics. (**A**) Histogram of confidence scores for the 522 618 human binary interactions in ConsensusPathDB. *X*-axis: confidence score in bins of 0.1; *Y*-axis: number of interactions. (**B**) Histogram of shortest path lengths connecting pairs of nodes in the PPI. (**C**) Node degree distribution of the PPI in log–log scale. *X*-axis: node degree; *Y*-axis: number of nodes. Graphs (B) and (C) were generated with the network analysis function (60) within the Cytoscape software (61). (**D**) Scatter plot of degree (*X*-axis) and core (*Y*-axis) of all 19 610 nodes in the PPI. (**E**) Box plot of node core distribution of 3347 recently annotated cancer genes from the Network of Cancer Genes, NCG version 7 (orange) and 3347 randomly selected genes. The *P*-value corresponds to the unpaired Wilcoxon’s rank sum test.

ConsensusPathDB contains a large integrated PPI network comprising 616 304 human interactions (Table 1). Of these, 522 618 are binary interactions composed of exactly two interaction partners; the rest are self-interactions or complex interactions comprising three or more partners. Similar to previous versions, all binary interactions have a numerical score assigned (range [0, 1]; Figure 2A). Scores have been computed by integrating several annotation-based and topology-based measures that quantify the confidence associated with each given interaction (59). In the web interface, these scores are additionally visualized with a ‘traffic light’ icon (green: high confidence >0.95; orange: moderate confidence [0.5–0.95]; red: low confidence <0.5).

The integrated PPI network covers 19 610 different human proteins and consists of well-known hubs with very high node degrees >1000 (e.g. MYC with 1932 interactions or TP53 with 1281 interactions; Table 2) and a large number of 6601 proteins with <10 interactions. We analyzed this network further using the network analysis function (60) within the Cytoscape software (61). As typical for biological networks, the node degree follows a power law and exhibits a small-world property with a median shortest path of 3 connecting two proteins (Figure 2B and C).

**Table 2.** Top 30 hub proteins in ConsensusPathDB 2022

Protein	Gene symbol	Node degree	Node core	Cancer gene (NCG V7)
PKHA4_HUMAN	PLEKHA4	2932	92	No
A4_HUMAN	APP	2554	92	No
ESR2_HUMAN	ESR2	2296	92	No
ESR1_HUMAN	ESR1	2200	92	Yes
NTRK1_HUMAN	NTRK1	1958	92	Yes
MYC_HUMAN	MYC	1932	92	Yes
KIF14_HUMAN	KIF14	1707	92	No
H4_HUMAN	H4C1	1685	92	No
JUN_HUMAN	JUN	1580	92	Yes
EGFR_HUMAN	EGFR	1436	92	Yes
CTRO_HUMAN	CIT	1383	92	No
NR2C2_HUMAN	NR2C2	1358	92	No
RECQ4_HUMAN	RECQL4	1353	92	Yes
BRD4_HUMAN	BRD4	1345	92	Yes
U5S1_HUMAN	EFTUD2	1345	92	Yes
RNF4_HUMAN	RNF4	1331	92	Yes
BIRC3_HUMAN	BIRC3	1324	92	Yes
UBC_HUMAN	UBC	1324	92	No
XPO1_HUMAN	XPO1	1310	92	Yes
P53_HUMAN	TP53	1281	92	Yes
EGLN3_HUMAN	EGLN3	1279	92	No
CUL3_HUMAN	CUL3	1229	92	Yes
BRCA1_HUMAN	BRCA1	1096	92	Yes
TIF1B_HUMAN	TRIM28	1085	92	Yes
GRB2_HUMAN	GRB2	1056	92	Yes
HD_HUMAN	HTT	1036	92	No
PHB_HUMAN	PHB	1017	92	No
KI20A_HUMAN	KIF20A	999	92	No
HSP7C_HUMAN	HSPA8	994	92	No
CSN5_HUMAN	COPS5	985	92	No

### The PPI as a resource for network propagation

Network propagation is a theoretical framework for network analyses. It describes a set of analysis tools that use experimental data such as genotype data, expression data or categorical data to initialize node weights and subsequently distribute these weights simultaneously to the network neighborhoods of the nodes (62). This process converges to a steady state and leads to a re-ranking of the original network nodes. This re-ranking typically amplifies functional associations and is used to identify hotspot subnetworks that agglomerate much of the experimental weights and can be associated with specific biological pathways or parts thereof. Typical applications are to draw inference on genotype–phenotype relations from mutation data (63) or to identify functional networks from gene and protein expression data (64). The integrated ConsensusPathDB PPI network is available from the download section of the web server. It has been used in the past as a resource for network propagation (8), and it has been found as one of the best-performing networks for disease gene identification in an independent benchmark comparison among 21 publicly available networks (7).

### Degree bias in PPI networks

It should be noted that the ConsensusPathDB PPI network, as many others, contains well-studied protein as hubs (Table 2), which may interfere with network inferences that are based on degree distributions because hubs typically gain a lot of weight in the propagation process because they are highly connected. There are two main biases in PPI networks associated with such hubs: experimental bias and annotation bias. Experimental bias is induced by the way interactions are measured, e.g. Y2H (65), because these experiments generate star-like structures in interaction graphs with the bait protein as center and prey proteins being connected with the center hub but usually not among themselves. Annotation bias is introduced by the trend to study interactions of already well-studied proteins, which attributes additional links to these hubs and leads to bias in the degree distribution of the PPI (66).

Node degree bias in network propagation can be reduced by either better controlling the hubs in the propagation step or taking into account more robust metrics in the re-ranking process. For this purpose, we have developed the network propagation method NetCore (67). NetCore uses the node core as an alternative node property instead of node degree to conduct the propagation of the experimental weights, which has been found to be more robust against the influence of hubs. Coreness, in contrast to degree, reflects the connectedness of the entire node environment rather than the center hub and thus downweights star-like structures. It is used for identification of influential nodes, i.e. nodes in the core of a network, in contrast to nodes in the periphery of the network. It has been shown that degree and core can be viewed mathematically as start and convergence states of a series of node operators called *H*-indices (68).

Although node core is more robust than node degree, both metrics are correlated (Figure 2D). In fact, it can be seen that most hubs (Table 2) are in the very inner core of the network and that higher core genes are typically disease genes that are well annotated. To exemplify this, we have investigated the core distribution of 3347 cancer genes and putative cancer genes as identified recently with the network of cancer genes, NCG version 7 (69). This core distribution is significantly higher than that of 3347 randomly chosen genes (Figure 2E; *P* = 3.25e−55), which reflects the fact that cancer genes are very influential in the PPI network, on the one hand, because they are intensively studied and, on the other hand, because they are highly connected and participate at multiple cellular processes.

## CONCLUSION

Through the integration of 31 human public interaction/pathway resources, ConsensusPathDB assembles one of the most comprehensive available maps of human interactions and pathways. Viewing and analyzing molecular data in the context of heterogeneous interactions allows detecting cellular mechanisms across annotation domains, which is essential in the interpretation of contemporary types of complex high-throughput data. The content increase updates the system with the most widely used interaction databases and the inclusion of regulatory gene sets for data interpretation opens a novel path for functional analysis and interpretation of gene lists. Additionally, the PPI network provides a valuable resource for network biology.

## DATA AVAILABILITY

ConsensusPathDB is freely accessible through the web server at http://consensuspathdb.org. All agglomerated interactions and pathway gene sets can be downloaded in the download section.

## Supplementary Material

gkab1128_Supplemental_Files
