# Supplementary material for: ConsensusPathDB 2022: molecular interactions update as a resource for network biology
Source: Nucleic Acids Res. 2021 Nov 25;50(D1):D587–95. doi: 10.1093/nar/gkab1128 (PMC8728246; doi:10.1093/nar/gkab1128)
Supplement: gkab1128_Supplemental_Files [file gkab1128_supplemental_files.zip › SupplementaryMaterial.pdf]

ConsensusPathDB 2022 - molecular interactions update as a resource for network biology

Kamburov Atanas[1], Herwig Ralf[2,*]

[1] R&D Digital Technologies Department, Bayer AG, Berlin, 13353, Germany
[2] Department of Computational Molecular Biology, Max-Planck-Institute for Molecular Genetics, Berlin, 14195, Germany

* To whom correspondence should be addressed. Tel: +49 30 84131587; Fax: +49 30 84131552; Email: herwig@molgen.mpg.de

Supplementary Material

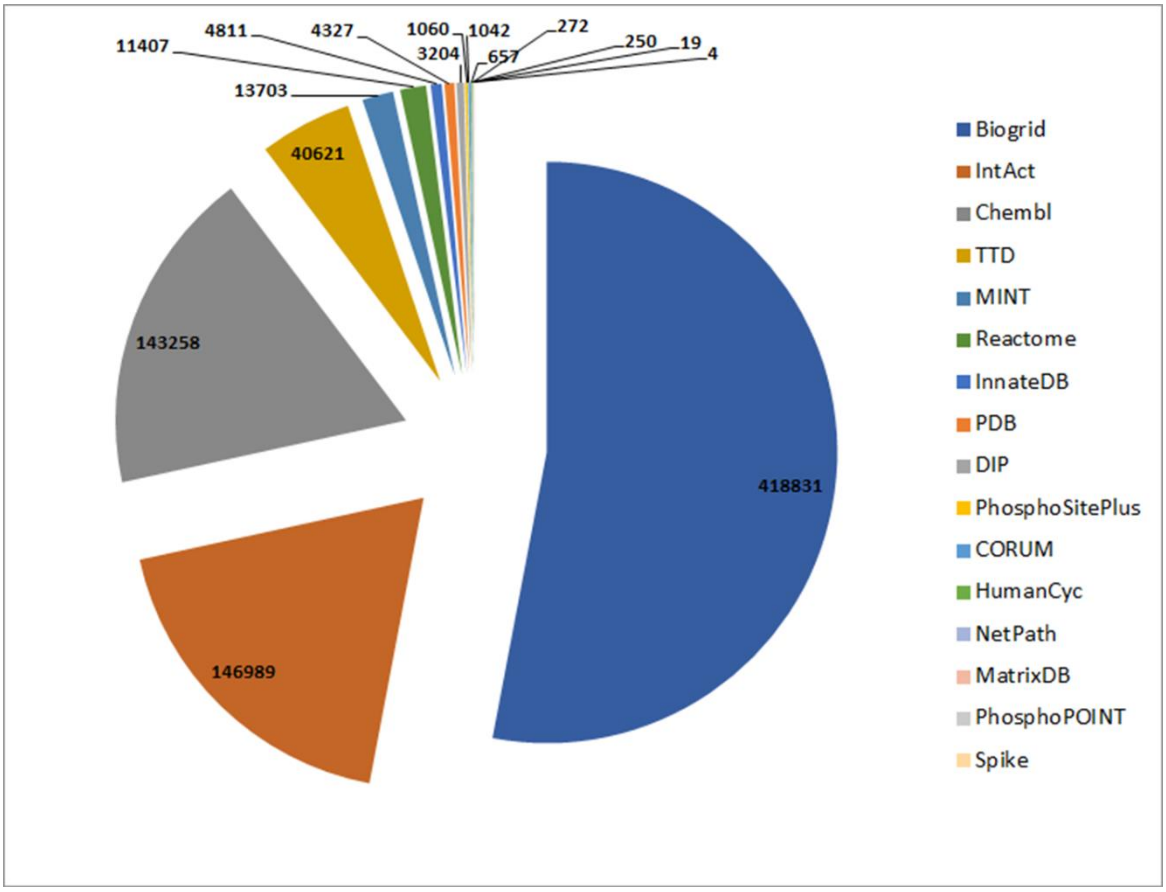

Supplementary Figure S1. Contributions of databases to the novel content of ConsensusPathDB. Listed are only databases with an increase in interactions since the last publication of ConsensusPathDB in 2013 (version 25). Numbers show the novel interactions.

Supplementary Table S1: Growth figures describing the increase in content of A) mouse and B) yeast interactions with respect to the last database publication in 2013 (5).

A	Mouse		
**Interaction type**	**2012 - Version 7 (# interactions)**	**2022 - Version 11 (# interactions)**	**Content growth (# interactions)**
protein-protein	16,561	75,823	59,262
signaling or metabolic	5,849	11,064	5,215
gene regulatory	33	3,193	3,160
genetic	0	516	516
drug-target	0	0	0

B	Yeast		
**Interaction type**	**2012 - Version 7 (# interactions)**	**2022 - Version 11 (# interactions)**	**Content growth (# interactions)**
protein-protein	259,031	240,208	-18,823
signaling or metabolic	2,194	3,826	1,632
gene regulatory	0	390	390
genetic	0	401,317	401,317
drug-target	0	0	0
